# Delivering HIV Gagp24 to DCIR Induces Strong Antibody Responses *In Vivo*

**DOI:** 10.1371/journal.pone.0135513

**Published:** 2015-09-25

**Authors:** Anne-Laure Flamar, Vanessa Contreras, Sandra Zurawski, Monica Montes, Nathatlie Dereuddre-Bosquet, Frédéric Martinon, Jacques Banchereau, Roger Le Grand, Gerard Zurawski, Yves Levy

**Affiliations:** 1 Baylor Institute for Immunology Research, Dallas, Texas, United States of America; 2 Vaccine Research Institute, Créteil, France; 3 INSERM UMR 955, Equipe 16, Créteil, France; 4 CEA, Division of Immuno-Virology, IDMIT Center, Institute for Emerging Diseases and Innovative Therapies, Fontenay-aux-Roses, France; 5 INSERM UMR 1184, Fontenay-aux-Roses, France; 6 Université Paris-Sud 11, Orsay, France; 7 Jackson Laboratory for Genomic Medicine, Farmington, Connecticut, United States of America; 8 Assistance Publique-Hôpitaux de Paris, Groupe Henri-Mondor Albert-Chenevier, service d’immunologie clinique, Créteil, France; 9 Université Paris Est, Faculté de médecine, Créteil, France; Emory University School of Medicine, UNITED STATES

## Abstract

Targeting dendritic cell-specific endocytic receptors using monoclonal antibodies fused to desired antigens is an approach widely used in vaccine development to enhance the poor immunogenicity of protein-based vaccines and to induce immune responses. Here, we engineered an anti-human DCIR recombinant antibody, which cross-reacts with the homologous cynomolgous macaque receptor and was fused via the heavy chain C-terminus to HIV Gagp24 protein (αDCIR.Gagp24). *In vitro*, αDCIR.Gagp24 expanded multifunctional antigen-specific memory CD4^+^ T cells recognizing multiple Gagp24 peptides from HIV-infected patient peripheral blood mononuclear cells. In non human primates, priming with αDCIR.Gagp24 without adjuvant elicited a strong anti-Gagp24 antibody response after the second immunization, while in the non-targeted HIV Gagp24 protein control groups the titers were weak. The presence of the double-stranded RNA poly(I:C) adjuvant significantly enhanced the anti-Gagp24 antibody response in all the groups and reduced the discrimination between the different vaccine groups. The avidity of the anti-Gagp24 antibody responses was similar with either αDCIR.Gagp24 or Gagp24 immunization, but increased from medium to high avidity in both groups when poly(I:C) was co-administered. This data provides a comparative analysis of DC-targeted and non-targeted proteins for their capacity to induce antigen-specific antibody responses *in vivo*. This study supports the further development of DCIR-based DC-targeting vaccines for protective durable antibody induction, especially in the absence of adjuvant.

## Introduction

Targeting antigen directly to antigen-presenting cells (APCs), such as dendritic cells (DCs), using recombinant antibodies (rAb) against APC-specific surface receptors fused to desired antigens is a way to increase immunogenicity of protein vaccines and reduce their effective dose. In most DC-targeting studies in mice, the vaccines were administered with a DC-activating agent or an adjuvant to induce potent CD8^+^ T cell responses [1–4], although this does not always seem to be necessary for generating antibody responses [5–9]. Poly(I:C), a synthetic double-stranded RNA, has been used as adjuvant with an anti-DEC-205 antibody for inducing antibody responses in non-human primates (NHPs) against a malaria circumsporozoite protein [10] and an human immunodeficiency type 1 virus (HIV-1) Gagp24 (Gagp24) protein [11].

Currently, most vaccines generate protection mainly through induction of antibodies. Studies have shown that the response to Gag is important in the effective cellular immune response to HIV-1 infection, supporting the rational that HIV-1 Gag is an essential HIV vaccine component [12–14]. These studies demonstrated that Gag-specific responses were the dominant CD4^+^ T cell responses to HIV in infected individuals [13]. HIV progressor patients generally have reduced ability to develop an antibody response to Gagp24 antigens, suggesting that delay of clinical manifestations of AIDS may be related to the presence of high levels of Gagp24-specific antibodies [15–17]. Therefore, there is still a need for optimizing immunity by improving adjuvants, vaccine delivery or both. This will hopefully lead in increased durability of cellular and humoral immunity to achieve protection.

In this study, we investigated the effect of delivering HIV Gagp24 protein fused to the heavy chain C-terminus of a recombinant Ab cross-reacting with both human and cynomolgus macaque dendritic cell immunoreceptor (DCIR) [18]. DCIR is expressed on all human APCs and was shown to mediate cross-priming [19] and antigen-presentation to CD4^+^ T cells [20]. It is also expressed on monocytes and moderately on isolated epidermal cells from cynomolgus macaques [18]. We show that HIV Gagp24 delivered to APCs in vitro via DCIR activates multifunctional antigen-specific memory CD4^+^ T cells from HIV-infected individuals. Although, it is well established in animal models that anti-HIV Gagp24 antibodies do not associate with vaccine-induced protection, here we used HIV Gagp24 as model antigen to assess HIV vaccine-induced antibody responses in vivo. We compare the magnitude of the HIV Gagp24 antibody responses following vaccination in NHPs with or without poIy(I:C) as adjuvant. These data show that targeting antigens to DCIR is a promising means for inducing rapid and sustained antigen-specific antibody responses without the need for adjuvant in the context of both prophylactic and therapeutic vaccines strategies.

## Results

### Production of anti-DCIR.Gagp24 fusion protein

**Fig 1:**
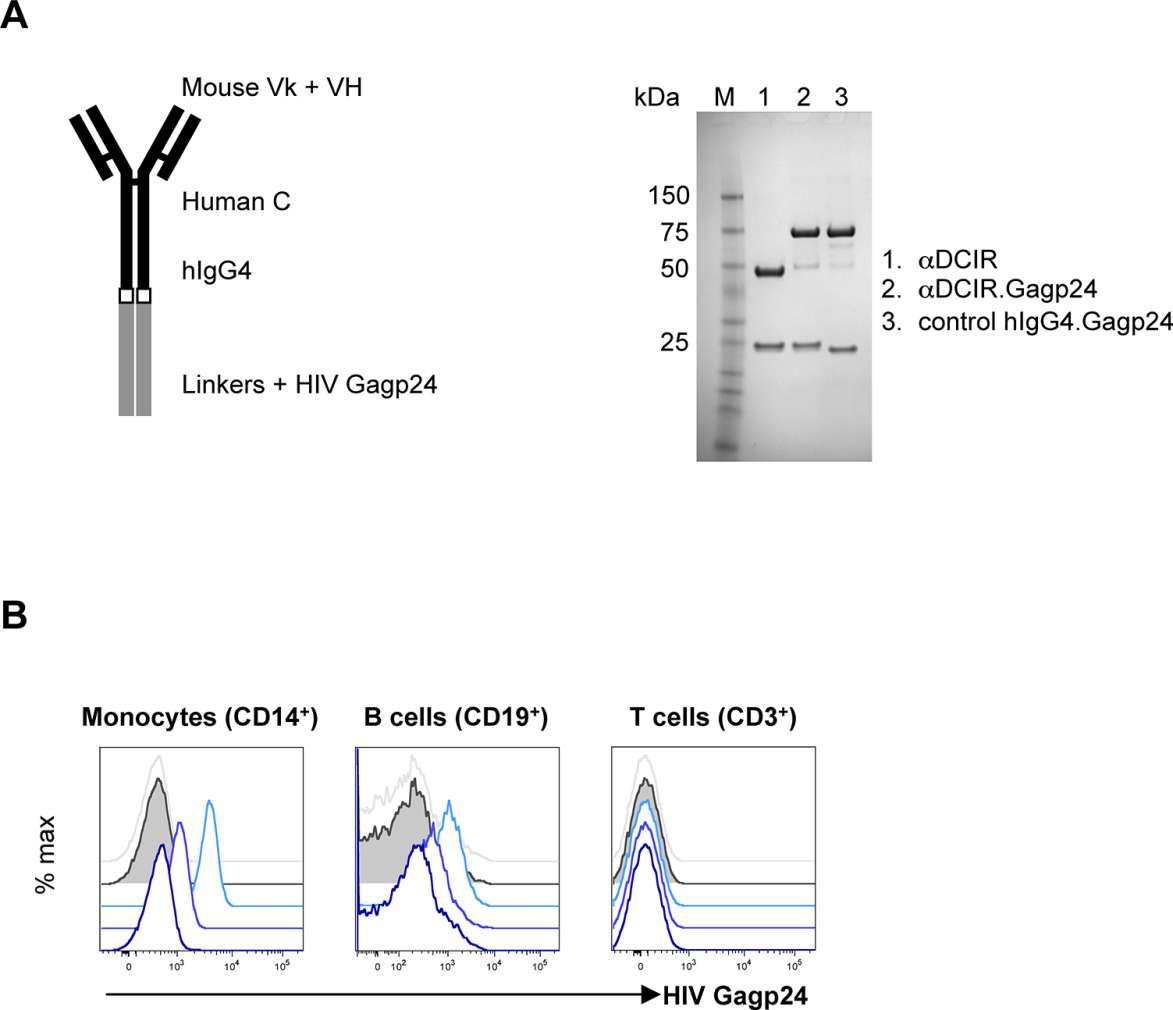
Characterization of the αDCIR.Gagp24 fusion rAbs. **A.** Schematic representation and SDS-PAGE analysis under reducing conditions of the αDCIR.Gagp24 fusion protein. Expression constructs for chimeric mouse variable (V) region-human IgG4 constant (C) region αDCIR and isotype control recombinant antibodies (rAbs) were engineered with the HIV Gagp24 coding region fused in frame to the heavy (H) chain C-terminus (left panel). These constructs were co-transfected with a matching light (k) chain expression vector into stable CHO-S cell lines and the secreted fusion rAbs were purified from the culture supernatants by protein A-affinity chromatography and analyzed on reducing SDS-PAGE. Proteins were stained with Coomassie Blue (right panel). **B.** Specific binding of αDCIR.Gagp24 rAb. Monocytes (left panel), B cells (middle panel) and T cells (right panel) from an HIV-infected patient PBMCs were treated with 3 nM, 0.3 nM and 30 pM of αDCIR.Gagp24 and 3 nM hIgG4.Gagp24 fusion proteins, followed by incubation with an anti-HIV Gagp24 PE-conjugated antibody and then analyzed by flow cytometry. Blue traces are staining from αDCIR.Gagp24. The blue color gradient represents the different concentrations from light blue (3 nM) to dark blue (30 pM). Grey traces (staining by 3 nM hIgG4.Gagp24) and grey solid traces (untreated cells) are the background staining. Data are representative of 2 different patients.

To generate human anti-DCIR.Gagp24 fusion protein, plasmid constructs directing the expression in mammalian cells of a secreted recombinant chimeric mouse anti-human DCIR receptor recombinant antibody (rAb) fused via the heavy chain C-terminus to HIV Gagp24 protein (αDCIR.Gagp24) were engineered as described [19]. A non-binding isotype matched control IgG4 [21] was also engineered as a negative control (hIgG4.Gagp24) (Fig 1A, left panel). The resulting αDCIR.Gagp24 and hIgG4.Gagp24 rAbs secreted from stable CHO-S cell lines were purified by protein A affinity (Fig 1A, right panel). As shown by detection of cell surface HIV Gagp24 antigen, αDCIR.Gagp24 rAb, but not hIgG4.Gagp24 specifically binds to DCIR on HIV-infected patient monocytes and B cells, but not to T cells (Fig 1B).

### HIV Gagp24 targeted to DCIR is efficiently presented to human CD4^+^ T cells *in vitro*

**Fig 2:**
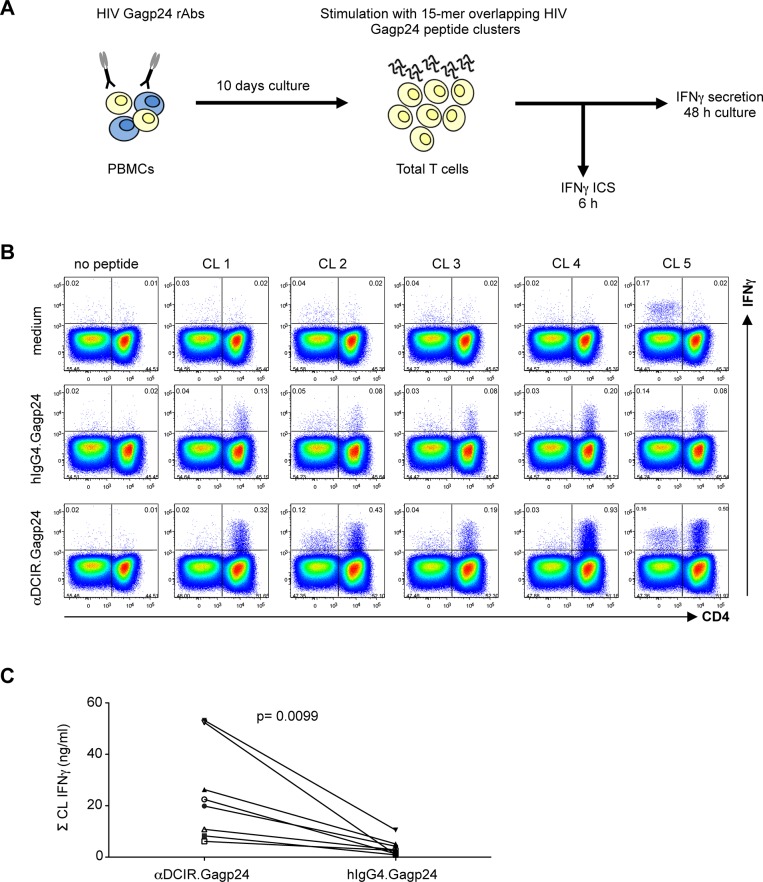
Low doses of αDCIR.Gagp24 expand a broad repertoire of HIV Gagp24-specific T cells. **A.** Experimental procedure. ICS, intracellular staining. **B.** PBMCs from an HIV-infected patient (patient A15) were cultured for 10 days in medium alone (top panel), or with 0.3 nM hIgG4.Gagp24 (middle panel), or 0.3 nM αDCIR.Gagp24 (lower panel) and then rechallenged for 6 h with or without (-C) 5 clusters (CL) of 15-mer overlapping peptides covering HIV Gagp24 in the presence of brefeldin A prior to intracellular IFNγ staining. The flow cytometry profiles are gated on the overall CD3^+^ T cell population. Data are representative of 4 different patients. **C.** PBMCs from 4 HIV-infected patients (patient A1, A2. A12 and A15) were cultured for 10 days with 0.3 nM (filed symbols) or 30 pM (open symbols) of αDCIR.Gagp24 or hIgG4.Gagp24 and then restimulated for 48 hrs with 5 clusters (CL) of 15-mer overlapping peptides covering HIV Gagp24. The culture supernatants were then harvested and IFNγ produced by total T cells was analyzed by multiplex bead-based assay. Each patient is represented by a different symbol. Data are presented as mean of sum of HIV Gagp24 clusters. Differences between αDCIR.Gagp24 and hIgG4.Gagp24 were assessed by a matched paired t-test.

To evaluate the efficacy of presentation of αDCIR.Gagp24 rAb, PBMCs from an HIV-infected patient were cultured for 10 days with increasing doses of αDCIR.Gagp24 or isotype control hIgG4.Gagp24 and IFNγ secretion was measured after restimulation with pools of 15-mer peptides spanning the HIV Gagp24 protein sequence (Fig 2A). We found that HIV Gagp24 protein was presented through DCIR leading to expansion of memory T cells specific to multiple HIV Gagp24 epitopes (S1 Fig). The isotype control also presented HIV Gagp24 protein to HIV-specific T cells but this presentation was restricted to > 10–100-fold higher doses (S1 Fig). Next, we assayed the type of T cells that were expanded by αDCIR.Gagp24 rAb. We incubated PBMCs from an HIV-infected patient (patient A15) with 0.3 nM of αDCIR.Gagp24 or hIgG4.Gagp24 for 10 days and then performed intracellular IFNγ staining after rechallenge with pools of HIV Gagp24 peptides (Fig 2A and 2B). Although, we did not detect any CD8^+^ T cell responses, CD4^+^ T cells produced IFNγ in response to HIV Gagp24 antigen (Fig 2B). In this particular patient, αDCIR.Gagp24 expanded CD4^+^ T cells specific to all 5 HIV Gagp24 regions we screened (Fig 2B). Moreover, αDCIR.Gagp24 was more efficient at expanding Gagp24-specific T cells producing IFNγ than the isotype control hIgG4.HIV Gagp24 given at the same concentrations (Fig 2B and 2C). Also, in the condition where the cells were not treated with the HIV Gagp24 fusion proteins but were restimulated with pools of HIV Gagp24 peptides, we could detect some expansion of peptide-specific T cells, which is expected in the HIV-infected patient population. In similar experiments with PBMCs from 2 healthy volunteers, no HIV peptide-specific T cells were detected in response to stimulation with αDCIR.Gagp24 (data not shown). Together these results indicate that a low concentration of αDCIR.Gagp24 rAb efficiently mediates presentation via DCIR delivery of multiple epitopes within the HIV Gagp24 protein to HIV-specific CD4^+^ memory T cells.

### Anti-DCIR.Gagp24 generates multifunctional memory CD4^+^ T cells to multiple HIV Gagp24 epitopes *in vitro*

**Fig 3:**
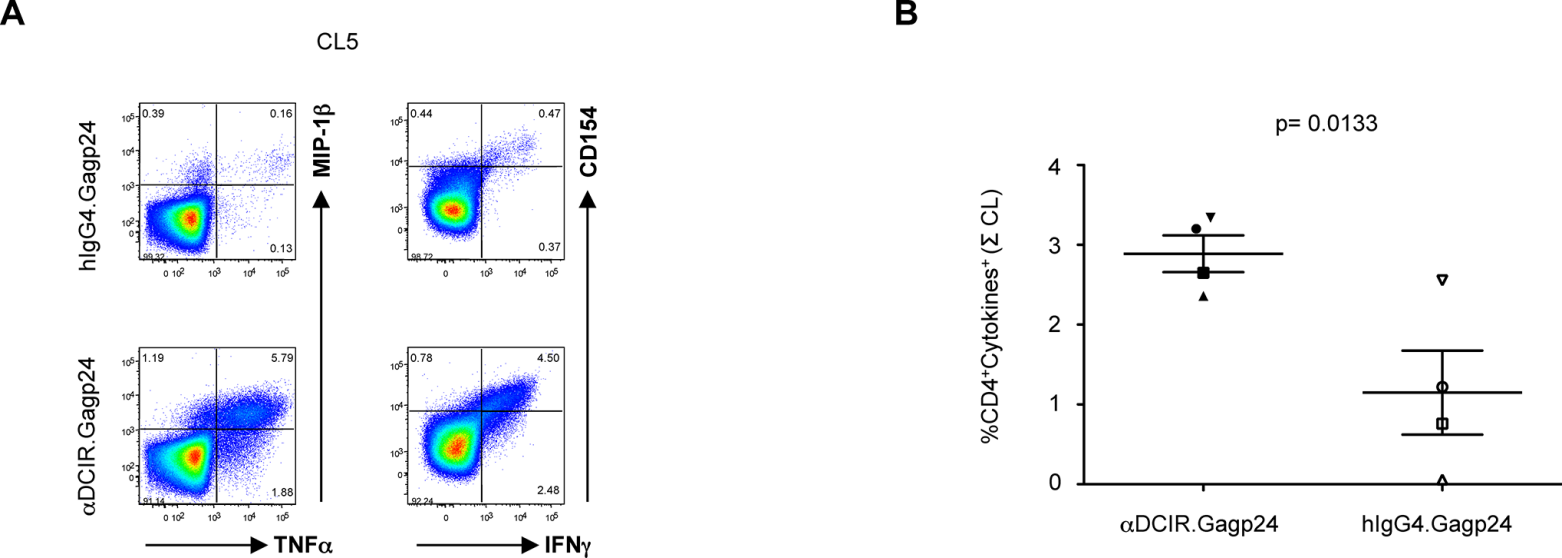
αDCIR.Gagp24 expands multifunctional HIV Gagp24-specific CD4^+^ T cells *in vitro*. PBMCs from HIV-infected patients (panel **A**, patient A15; panel **B**, patients A2, A1, A15) were cultured for 10 days with 0.3 nM of αDCIR.Gagp24 or hIgG4.Gag p24 or left unstimulated and then restimulated for 6 hrs with or without clusters of 15-mer overlapping peptides covering HIV Gagp24 in the presence of brefeldin A prior to intracellular cytokine staining. The profiles are gated on the overall CD4^+^ T cell populations, with the percentages of populations responding to the designated peptides shown in each plot. **A**. Coordinate analysis of TNFα versus MIP-1β, IFNγ or CD154 expression (patient A15). Data are representative of 4 different patients. **B**. Multifunctionality of the cytokine responses. Combined percentages of 5 CD4^+^ T cell populations producing different combinations of at least 3 cytokines (IFNγ, TNFα, MIP-1β or CD154). Data are presented as mean of sum of HIV Gagp24 clusters ± SEM for 3 patients. Differences between αDCIR.Gagp24 and hIgG4.Gagp24 were assessed by a matched paired t-test.

To further characterize the HIV-specific CD4^+^ T cells expanded with αDCIR.Gagp24 rAb, we looked at the antigen-specific cytokine and chemokine production profiles of these T cells after restimulation with clusters of HIV Gagp24 peptides. Thus, in PBMC cultures from one representative HIV-infected patient, most of the HIV-specific-CD4^+^ T cells stimulated with αDCIR.Gagp24 rAb for 10 days co-expressed intracellular IFNγ, TNFα, MIP-1β and CD154 (CD40 ligand), in response to stimulation with HIV Gagp24 peptide clusters (Fig 3A). This expansion of HIV Gagp24-specific T cells producing multiple cytokines and chemokines was peptide-specific and strongly enhanced by stimulation with αDCIR.Gagp24 rAb compared to stimulation with the isotype control hIgG4.Gagp24 (Fig 3A and 3B). Overall, these results show that αDCIR.Gagp24 rAb expands multifunctional HIV peptide-specific memory CD4^+^ T cells against a broad range of HIV Gagp24 epitopes.

### Immunization with anti-DCIR.Gagp24 without adjuvant induces strong Gagp24-specific antibody responses in NHPs

**Fig 4:**
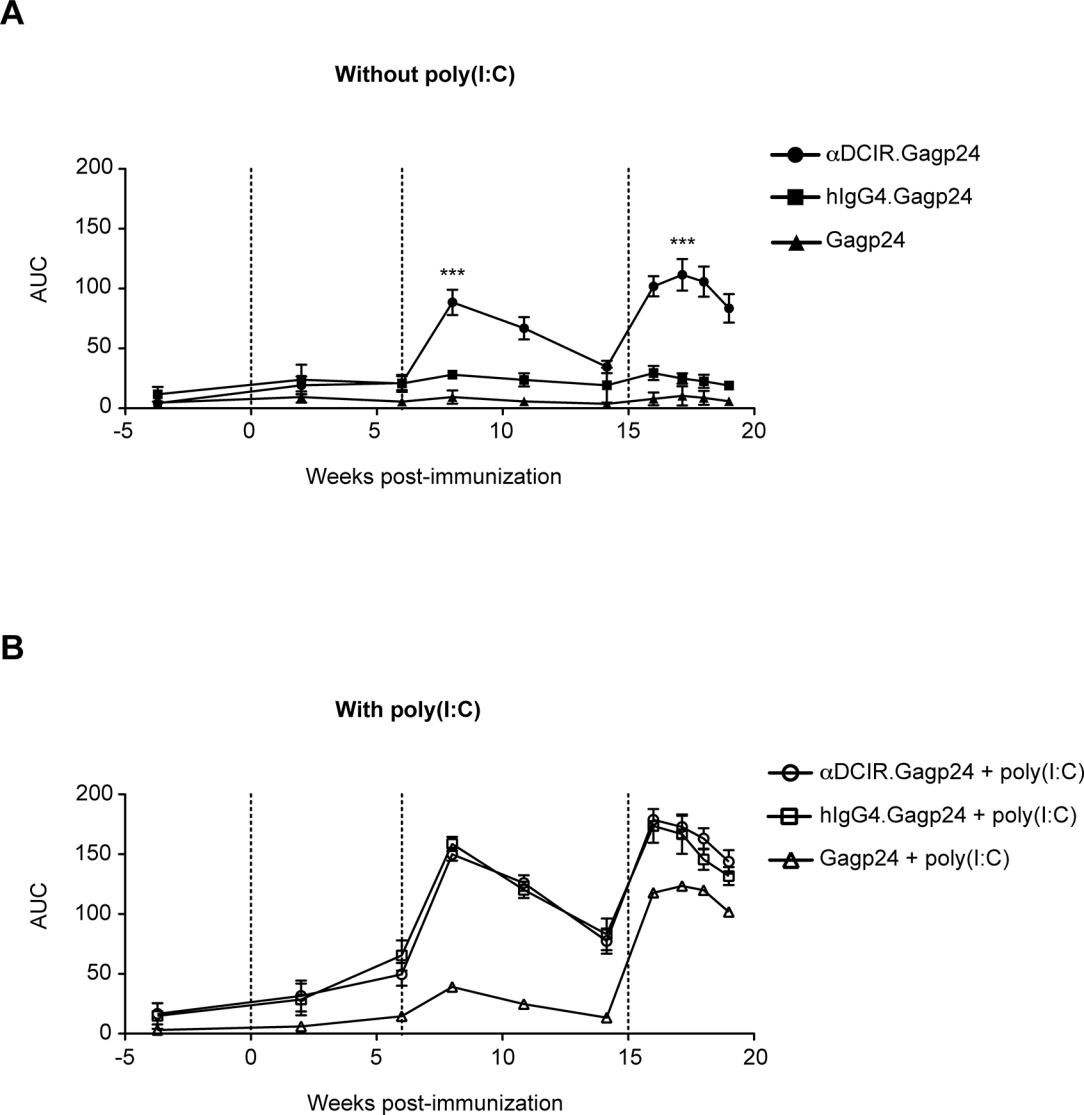
Immunization with αDCIR.Gagp24 without adjuvant generates high titers of HIV Gagp24 antibody responses in NHPs. Animals were immunized i.d. three times at week 0, 6 and 15 with αDCIR.Gag24 or control hIgG4.Gagp24 or the molar equivalent of the HIV Gagp24 protein with or without poly(I:C). HIV Gagp24-specific IgG antibodies in serum were assessed at indicated time points post-immunization with αDCIR.Gagp24 or hIgG4.Gagp24 or Gagp24 without adjuvant **(A)** or with poly(I:C) **(B)**. Dashed lines indicates times of immunization. The serial measurements of Gagp24-specific antibody responses collected on individual monkeys over time among the vaccine groups were summarized by calculating the area under the titration curves for each monkey at each time point, and longitudinal differences between the vaccine groups were assessed by linear mixed model analysis. Data are presented as mean ± SEM. *, p<0.01; **, p<0.001; ***, p<0.0001 compared with hIgG4.Gagp24 and Gagp24 without poly(I:C).

To assess the capacity of αDCIR.Gagp24 rAb to induce Gagp24-specific humoral immune responses in vivo, we immunized cynomolgous macaques intradermally (i.d.) with αDCIR.Gagp24, hIgG4.Gagp24 rAbs or Gagp24 protein with or without poly(I:C) as adjuvant (Table 1). In vivo analysis of B cell responses was performed since in vitro culture systems used to assess antibody are complex. They require expansion and conversion of memory B cells into antibody-secreting cells by in vitro culture using several different stimuli, including Toll-like receptor ligands, pokeweed mitogen, cytokine cocktails, CD40 ligation, and B cell receptor crosslinking [22]. Furthermore, the analysis of antigen-specific human memory B cells has been challenging because they circulate at very low frequencies in peripheral blood, do not secrete antibodies, and proliferate only at a very slow rate under steady-state conditions. The magnitude of the HIV Gagp24 antibody response in serum from immunized macaques was assessed during the course of the study by ELISA. In the absence of adjuvant, serum anti-Gagp24 antibodies were low but detectable 2 weeks after priming (week 2) in all the two groups receiving rAb.Gagp24 (Fig 4A and S2A Fig). At 2 weeks following the first boost (week 8), limiting dilution analysis showed a ~16-fold increase in Gagp24-specific IgG antibody titers in the NHP group immunized with αDCIR.Gagp24 rAb, while titers in the groups that received hIgG4.Gagp24 rAb or Gagp24 were unchanged (S2A Fig). We observed in all the groups a small decline in the level of anti-Gagp24 antibody responses over the next 6 weeks (weeks 8 to 14) (Fig 4A). The third immunization with αDCIR.Gagp24 rAb boosted the titers a further 4-fold compared to the maximum peak response obtained at week 8, while the titers in the animals immunized with hIgG4.Gagp24 rAb or Gagp24 remained low (S2A Fig). These data show that αDCIR.Gagp24 rAb elicits a robust anti-Gagp24 antibody response in the absence of adjuvant in vivo compared to hIgG4.Gagp24 rAb or Gagp24 (p<0.005 in both groups from weeks 8 to 19).

**Table 1:** Immunization schedules used in this study.

Group (no. of monkeys)	Immunization (time point [week])
Group1 (6)	αDCIR.Gag24 (week 0; 6; 15)
Group2 (6)	αDCIR.Gag24+ poly(I:C) (week 0; 6; 15)
Group3 (3)	hIgG4.Gag24 (week 0; 6; 15)
Group4 (3)	hIgG4.Gag24+ poly(I:C) (week 0; 6; 15)
Group5 (3)	Gag24 (week 0; 6; 15)
Group6 (3)	Gag24 + poly(I:C) (wk 0; 6; 15)

**Fig 5:**
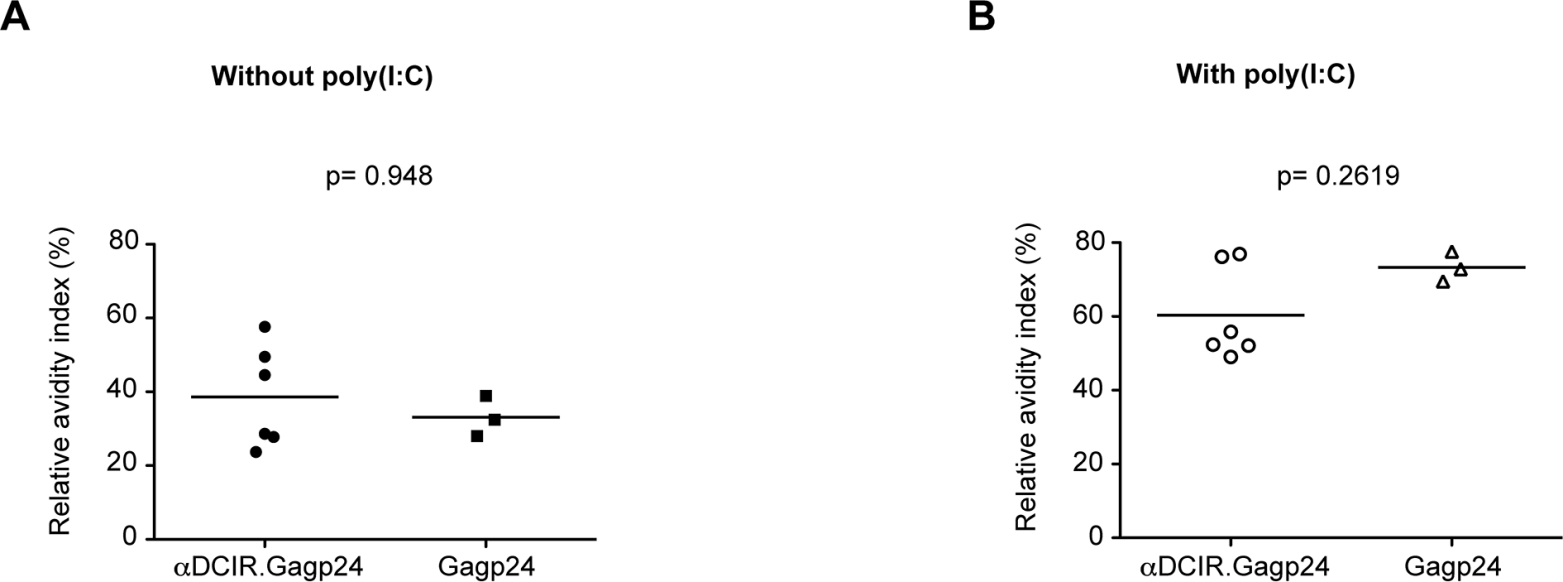
Anti-HIV Gagp24 antibody avidity following vaccination. Animals were immunized i.d. three times at week 0, 6 and 15 with αDCIR.Gag24 or the molar equivalent of the Gagp24 protein with or without poly(I:C). Serum HIV Gagp24-specific IgG antibody avidity was measured by ELISA 2 weeks after the third injection (week 17) with αDCIR.Gagp24 or Gagp24 without adjuvant **(A)** or with poly(I:C) **(B)**. Relative avidity index (RAI) (%) of Gagp24-specific IgG for the individual monkeys is presented.

In the presence of poly(I:C), anti-Gagp24 antibody titers 2 weeks after the first injection (week 2) were again low but detectable in the group that received αDCIR.Gagp24 and hIgG4.Gag p24 rAbs (Fig 4B and S2B Fig). After the first boost (week 8), NHPs immunized with αDCIR.Gagp24 or hIgG4.Gagp24 rAbs developed similar higher titers of anti-Gagp24 antibodies compared to the group that received Gagp24 (S2B Fig). However, after the third injection (week 17), the magnitude of Gagp24-specifc antibody response was further increased in all 3 groups to a similar level (Fig 4B and S2B Fig). These results indicate that poly(I:C) helps the development of antibody responses in vivo, but masks the discrimination between the different vaccine groups. To determine the quality of the anti-Gagp24 antibodies at the peak response (week 17), we performed ELISA in the presence of the dissociating agent ammonium thiocyanate in order to specifically disrupt lower affinity antigen–antibody binding and thus estimate the relative avidity of the Gagp24-specifc antibodies. Low to moderate avidity responses were induced by αDCIR.Gagp24 or Gagp24 without poly(I:C) (Fig 5A), whereas high avidity responses were observed in animals immunized with αDCIR.Gagp24 or Gagp24 with poly(I:C) (Fig 5B). Together, these data demonstrate that αDCIR.Gagp24 rAb induces a strong Gagp24-specific humoral immune response in vivo that can be further enhanced in the presence of adjuvant and this effect is sustained over time.

Animals were immunized i.d. three times with 250 μg αDCIR.Gag24 (n = 6) or control hIgG4.Gagp24 (n = 3) or the molar equivalent of the HIV Gagp24 protein (n = 3), with or without 250 μg poly(I:C).

## Discussion

Over the last decade, studies for improving prophylactic or therapeutic vaccines have focused on specifically delivering antigen to APCs through the linking of antigen to rAb against receptors expressed on APC surfaces. This strategy overcomes laborious ex vivo DC manipulation and reinjection into patients to induce antigen-specific immunity [23]. In this present study, we show that HIV Gagp24 protein when delivered to APCs in vitro through DCIR is efficiently processed and multiple peptide epitopes are presented resulting in expansion of HIV Gagp24-specific memory CD4^+^ T cells from PBMC cultures of HIV-infected individuals. Moreover, these expanded Gagp24-specific CD4^+^ T cells produce multiple cytokines in a peptide-specific manner. The isotype control has a reduced effect on antigen-specific CD4^+^ T cell expansion, which occurred only when high doses were used. In previous published studies, targeting HIV Gagp24 to the DEC-205 receptor in PBMC cultures from HIV-infected patients expanded antigen-specific CD8^+^ T cells, but no HIV Gagp24-specific CD4^+^ T cells were detected [24, 25]. Although the HIV-infected individuals we screened exhibited HIV Gagp24-specific CD8^+^ T cell responses that were too weak for us to study, another study reported that targeting HIV Gagp24 to DCIR in the presence of Toll-like receptor 7/8 ligands led to induction of antigen-specific CD8^+^ T cells from healthy individuals [19]. More recently, our group has reported targeting HIV epitopes in vitro, including Gagp24, Gap17, Nef and Pol, to CD40 receptor elicited memory CD4^+^ and CD8^+^ T cell responses [21]. Therefore, this supports the concept that depending on the cell surface receptor targeted in combination with different adjuvants, distinct types or qualities of immune responses might be generated, as previously observed by others [4, 7, 26, 27].

In this study, we also compare the immunogenicity of targeting HIV Gagp24 to DCIR versus delivering HIV Gagp24 in a non-receptor specific manner in vivo in NHPs. Remarkably, our data indicated that a single vaccination with αDCIR.Gagp24 rAb induced Gagp24-specific IgG production in vivo 2 weeks post-immunization in the absence of adjuvant or apparent sign of DC activation ([19] and unpublished data). The high Gagp24-specific antibody titers obtained after the second immunization with αDCIR.Gagp24 rAb were only slightly boosted by a third injection (p = 0.0625), but were further enhanced by co-injection with poly(I:C) (p<0.0001). This may be a consequence of the high antibody response to first boost being near maximal. Also, we cannot exclude that, with multiple immunizations, the antibodies generated in the primary injections, including antibodies against the human constant region or the mouse variable region of the αDCIR.Gagp24 antibody would not attenuate the response in the latter vaccinations. In another study where we measured the antibody responses against the targeting vaccine vehicle, they remained lower than the antigen-specific antibody responses even after the third vaccine injection [28]. This issue may also be overcome by delivering the antigen in different forms for the prime or the boost (e.g., DNA or viral vector vaccines). This approach could be used as a component of an effective vaccination strategy for generating some fast-responding memory B cells for future protection against that pathogen. In particular, the HIV Gagp24 antigen can be replaced with carefully designed HIV envelope glycoproteins, in order to induce anti-envelope antibodies with functions such as neutralization and antibody-dependent cell-mediated cytotoxicity, which are certainly highly relevant for vaccine efficacy.

Boosting with αDCIR.Gagp24 without adjuvant in vivo increased Gagp24-specific antibody responses compared to NHPs vaccinated with the isotype control hIgG4.Gagp24 and this difference was sustained over an 11-week period (weeks 8 to 19, p<0.005). However, the similar increased antibody responses observed in both groups in combination with poly(I:C) masks the specific effect of targeting through DCIR. We also noticed that, in the presence of poly(I:C), targeting HIV Gagp24 using an isotype control antibody led to induction of Gagp24-specific antibodies after the first boost that were of higher titers than when HIV Gagp24 protein was delivered alone. However, these differences were less pronounced, especially after the second boost. These antibody-Gagp24 fusion proteins are human IgG4 and were engineered with two additional mutations in the Fc region to abrogate residual human Fc receptor binding [29], however, we cannot rule out the possibility that human IgG4 interacts with the cynomolgous Fc receptor, or that hIgG4.Gagp24, because of its larger size, is intrinsically more immunogenic than the HIV Gagp24 protein alone. It may also be possible that the isotype control hIgG4.Gagp24 rAb is binding and being taken up by other cells in the dermis. To our knowledge, our study also reports for the first time the efficacy of intradermal vaccination of antibody-antigen DC-targeting reagents for induction of HIV-specific humoral immune responses. As previously reported, the human CD14^+^ dermal DCs expressing DCIR [19] can promote antibody production by B cells [30]. We also confirmed that poly(I:C) enhances antibody production in NHPs primed with DC-targeted vaccines [11]. In one study where NHPs were primed subcutaneously with αDEC-205.Gagp24 and poly(I:C) and boosted with recombinant vaccinia virus NYVAC, the avidity of the elicited Gagp24-specific antibodies was lower with the DEC-205-targeted vaccine than with the HIV Gagp24 protein alone [11]. Surprisingly, we found that the avidity of Gagp24-specific antibodies was within the same high avidity range in the NHPs vaccinated with αDCIR.Gagp24 plus poly(I:C) or Gagp24 plus poly(I:C). Therefore, additional NHP vaccine studies are needed to investigate in more detail different routes of delivery and the characteristics of the elicited antibody such as affinity, avidity, Ig isotype and potential cross-reactivity to different virus clades. Here we show that delivering αDCIR.Gagp24 in vivo resulted in enhanced antigen-specific antibody responses, most likely through specific targeting of DCIR, bypassing the prerequisite for adjuvant, and this offers potential advantages for safety reasons without the secondary effects of adjuvants. The antibody responses to the αDCIR.Gagp24 construct used herein was able to maintain intact B cells epitopes suggesting that this approach could be applied to other conformational antigens for which antibodies are known to mediate protection against infection (e.g., Influenza A PR8 hemagglutinin HA1 domain, unpublished data). In vivo studies will be undertaken to explore the capacity of αDCIR antibodies fused to HIV envelope proteins to elicit antigen-specific antibodies.

In conclusion, these findings provide a strong rationale for targeting antigens to DCIR in vivo for inducing strong antigen-specific antibody responses without the need for adjuvant and this may have important implications for enhancing vaccine efficacy to protect against many infectious diseases.

## Materials and Methods

### Cloning and production of recombinant anti-human DCIR antibody fused to HIV Gagp24

Mammalian cell vectors directing the secretion of chimeric mouse anti-human DCIR antibody variable regions fused to human IgG4 heavy (H) and light (L) chain constant regions were engineered as described in [19, 29]. The HIV Gagp24 linker is gi|119624034| MHC, class II, DR α residues 60–75 preceding gi|28872819| Gagp24 [HIV 1] residues 133–363 [29]. The Gagp24 coding region was then transferred to modified pIRES2-DsRed2 transient transfection vectors [19] or vectors for stable transfection of CHO-S cell lines for expression and subsequent purification of αDCIR.Gagp24 and isotype control hIgG4.Gagp24 using methods described in [27]. Endotoxin levels were 0.01 ng/mg for αDCIR.Gagp24, 0.09 ng/mg for hIgG4.Gagp24.

### Apheresis samples

Apheresis procedures were performed on highly active anti-retroviral therapy (HAART)-treated HIV-infected patients and healthy HIV-uninfected volunteers after written informed consent was collected. This protocol was reviewed and approved by the Baylor Research Institute Institutional Review Board. The HIV-infected patients enrolled in our study were not selected based on their CD4^+^ T cell counts (253–480 cells/μl) but they all controlled their viral load (HIV RNA viral load level < 50 copies/ml) under HAART.

### Preparation of cells

PBMCs were purified from leukapheresis blood samples and used after cryopreservation. PBMCs were enriched from the leukapheresis according to cellular density and size by elutriation (Elutra, CaridianBCT, Lakewood, CO) as per the manufacturer's recommendations.

### HIV Gagp24 peptides

Pools of 11 overlapping (staggered by 4 aa) 15-mer long HIV Gagp24 peptides (Bio-Synthesis, Lewisville, TX) were resuspended in DMSO (Sigma-Aldrich, St. Louis, MO).

### Cell culture and cytokine assay

PBMCs loaded with or without αDCIR.Gagp24 or isotype control rAbs were cultured for 10 days in complete RPMI 1640 media (Life Technologies, Carlsbad, CA) containing 10% human serum type AB (Gemini Bio-Products, West Sacramento, CA) at 37°C and 5% CO_2_. 100 IU/ml of IL-2 was added on day 2 and fresh media was added every 2 days. On day 10, cultures were restimulated with pools of overlapping 15-mer long HIV Gagp24 peptides at a final concentration of 2 μM each and analyzed after 6 h for cell surface markers and intracellular cytokine production as per the standard intracellular cytokine staining assay, or at 48 h for cytokine secretion into the culture supernatants. The secreted cytokines and chemokines were then measured in the culture supernatants using Luminex multiplex bead-based technology and a Bio-Plex 200 instrument (BioRad, Hercules, CA) according to the manufacturer’s instructions.

### Surface and intracellular staining

Briefly, cells were incubated for 6 h at 37°C in 5% CO_2_ with pools of overlapping 15-mer long peptides at a final concentration of 2 μM each, stimulated with SEB (Sigma) at 1 μg/ml or left unstimulated (negative control). The secretion inhibitor brefeldin A (Sigma) was added for the final 5 h of culture. After the 6 h incubation, surface staining for CD3-PercP, CD4-PECy7, CD8-PB (unless otherwise stated, staining reagents, instrumentation and software were from BD Biosciences) and live/dead fixable aqua dye (Life Technologies) was performed and then followed by intracellular staining for IFNγ-APC, TNFα-FITC, MIP-1β-PE, CD154-APC-eFluor780 (eBiosciences, San Diego, CA). Analysis was done on a two-laser CANTO II flow cytometer (BD Biosciences) and data analyzed with FlowJo software (TreeStar Inc., Ashland, OR). Spectral compensation was performed for each experiment with each individual mAb used in the surface and intracellular cytokines staining using compensation particles (BD Biosciences).

### Animals

Adult male cynomolgus macaques (Macaca fascicularis) (n = 24) imported from Mauritius and weighing 4 to 8 kg were housed at the CEA facility (Fontenay-aux-Roses, France; accreditation no.: B 92-032-02). All experiments were conducted according to the European guidelines for NHP care (EU Directive N 2010/63/EU; investigator accreditation no.: RLG, B 92–073; FM, C 92–241). All animals included in this study were previously confirmed negative for SIV, STLV, Herpes B virus, Filovirus, SRV-1, SRV-2 and measles. Animals were housed in groups, received standard primate feed and fresh fruit daily, and had ad libitum access to water. Cages also contained sources of environmental enrichment such as hiding places, hanging ropes and toys. Before each blood sampling and vaccine injection, animals were sedated with ketamine chlorohydrate (10–15 mg/kg, Rhone-Merieux, Lyon, France). Animal welfare was monitored daily by estimation of food consumption, weight changes and behavior. Animals were euthanized by sedation with ketamine followed by intravenous injection of a lethal dose of sodium pentobarbital. The Regional Animal Care and Use Committee (Comité Régional d’Ethique Ile-de-France Sud, reference 12–013) and the Baylor Research Institute Animal Care and Use Committee (reference A10-015) reviewed and approved this study.

### Immunizations

Animals were immunized i.d. three times with 250 μg αDCIR.Gag24 (n = 6) or control hIgG4.Gagp24 (n = 3) or the molar equivalent of the HIV Gagp24 protein (n = 3) (containing 63 μg of HIV Gagp24 protein), with or without 250 μg poly(I:C). All injections were given in a total volume of 1 ml (10 injections of 100 μL) in a single site i.d. at weeks 0, 6 and 15. High-molecular weight (HMW) poly(I:C) was purchased from InvivoGen (San Diego, CA). HIV Gagp24 protein was obtained from PX'Therapeutics (Grenoble, France).

### ELISA for anti-Gagp24 antibodies

HIV Gagp24-specific IgG antibody titers in serum from vaccinated animals were assessed at indicated time point post-immunization using ELISA. To determine Ag-specific Ab titers, ELISA plates were coated with 2 μg/ml Coh.Gagp24 [29] in 0.2 M sodium carbonate-bicarbonate buffer, pH 9.4. Serial dilutions of serum in 2.5% CBS-K ChemiBlock (Millipore, Billerica, MA) in Dulbecco's PBS (D-PBS, Life Technologies) were incubated in the wells overnight at 4°C. After washing, plates were incubated with HRP-conjugated goat anti-human IgG (Jackson ImmunoResearch, West Grove, PA) in TBS blocking solution (Thermo Scientific, Rockford, IL) for 2 h at 37°C, then washed and developed with HRP substrate (TMB, Life Technologies), stopped with equal volume of 1N HCl and read at 450 nm. The log_10_ transformed and normalized areas under the curves (AUC) data were calculated for each animal at each time point using GraphPad Prism 5 software (GraphPad, La Jolla, CA). For assessment of serum anti-Gagp24 antibody avidity, the anti-Gagp24 ELISA described above was adapted by incubating sera for 20 min at 37°C with 1 M ammonium thiocynate (NH4SCN) pH 6.0 as a dissociating agent to remove low-affinity antibodies as described [31]. The relative avidity index (RAI) was calculated by measurement of sample extinction after NH4SCN treatment, multiplied with factor 100 and divided by sample extinction without NH4SCN treatment and expressed in percentage (%). A RAI below 40% was considered of low-avidity antibodies, between 40% and 60% represents medium affinity antibodies, while a RAI over 60% is indicative of high-avidity antibodies [32].

### Statistical analyses

Statistical analyses of log_10_ transformed antibody AUC data were performed using SAS software (v9.3) (SAS Institute Inc., NC). A linear mixed model analysis, accounting for repeated measures and unequal time point spacing, was employed to test for significant differences in AUC between selected vaccination groups. Comparisons within the same groups of NHPs were assessed using the 2-tailed non-parametric Wilcoxon matched-pair test (GraphPad Prism 5). Comparisons between groups of NHPs were assessed using the 2-tailed non-parametric Mann-Whitney test (GraphPad Prism 5). P values of <0.01 were considered significant for all hypothesis testing.

## Supporting Information

S1 Fig Breadth of HIV Gag p24-specific T cell responses after stimulation with αDCIR.Gagp24. PBMCs from 4 HIV-infected patients (patients A1, A2, A12 and A15) were cultured for 10 days with a dose range from 30 pM to 3 nM of αDCIR.Gagp24 (blue bars, left panels) or hIgG4.Gag p24 (grey bars, right panels) or left unstimulated (unfilled bars) and restimulated for 48 hrs with or without (-C) 5 clusters (CL) of 15-mer overlapping peptides covering HIV Gagp24. The culture supernatants were then harvested and IFNγ secreted by total T cells was analyzed by multiplex bead-based assay. From left to right, concentrations are: 3 nM, 0.3 nM and 30 pM. Data are presented as mean ± SEM.
(TIF)

S2 Fig Serum anti-HIV Gagp24 antibody titers after vaccination. Animals were immunized i.d. three times at week 0, 6 and 15 with αDCIR.Gag24 or control hIgG4.Gagp24 or the molar equivalent of the Gagp24 protein with or without poly(I:C). HIV Gagp24-specific IgG antibody titers in serum were measured by ELISA at indicated time points post-immunization with αDCIR.Gagp24 or hIgG4.Gagp24 or Gagp24 without adjuvant **(A)** or with poly(I:C) **(B)**. Data are presented as mean ± SEM.
(TIF)
