# Supplementary figures and images for: Delivering HIV Gagp24 to DCIR Induces Strong Antibody Responses In Vivo
Source: PLoS One. 2015 Sep 25;10(9):e0135513. doi: 10.1371/journal.pone.0135513 (PMC4583231; doi:10.1371/journal.pone.0135513)

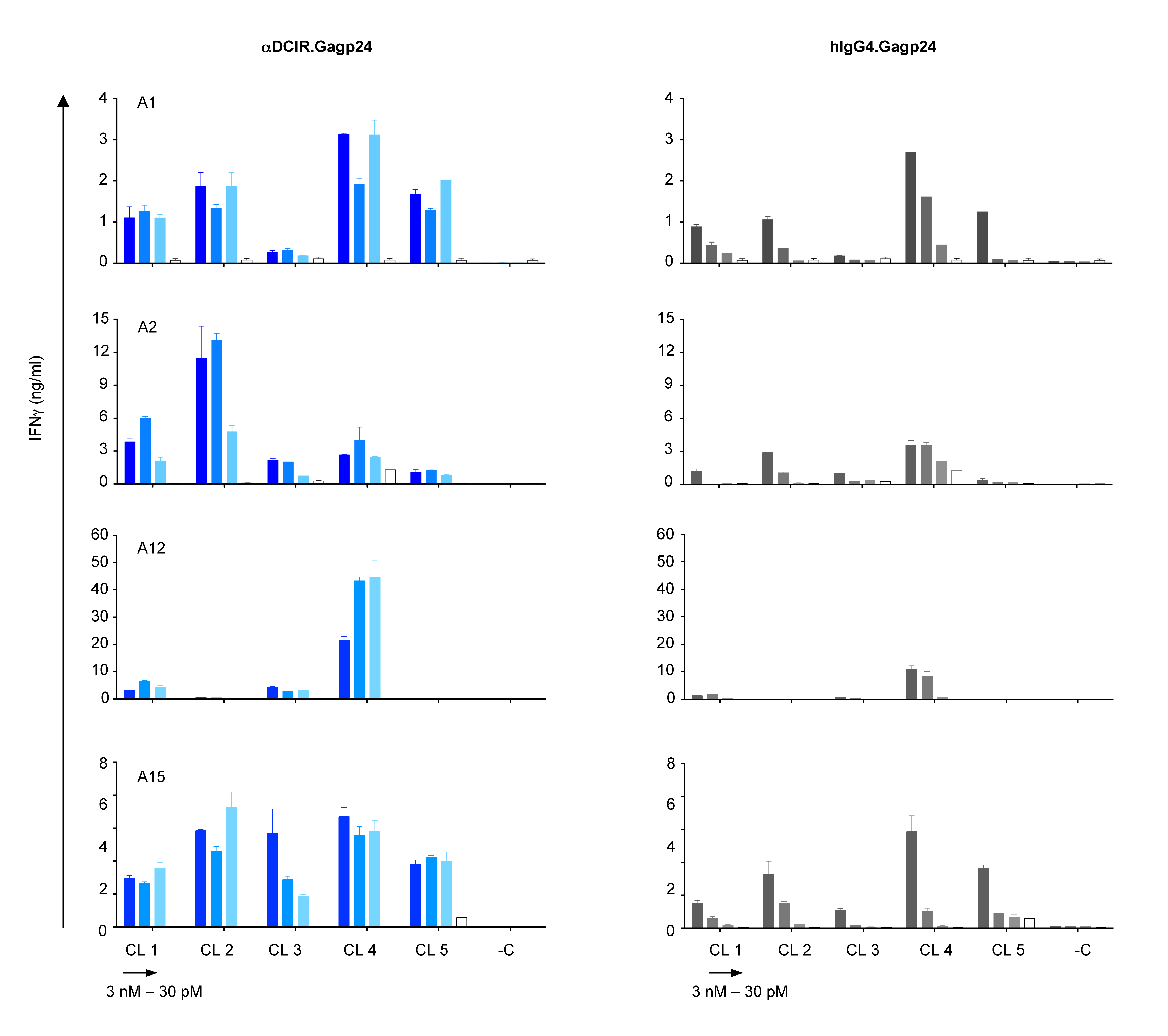

Supplement: S1 Fig — PBMCs from 4 HIV-infected patients (patients A1, A2, A12 and A15) were cultured for 10 days with a dose range from 30 pM to 3 nM of αDCIR.Gagp24 (blue bars, left panels) or hIgG4.Gag p24 (grey bars, right panels) or left unstimulated (unfilled bars) and restimulated for 48 hrs with or without (-C) 5 clusters (CL) of 15-mer overlapping peptides covering HIV Gagp24. The culture supernatants were then harvested and IFNγ secreted by total T cells was analyzed by multiplex bead-based assay. From left to right, concentrations are: 3 nM, 0.3 nM and 30 pM. Data are presented as mean ± SEM. (TIF) [file pone.0135513.s001.tif]

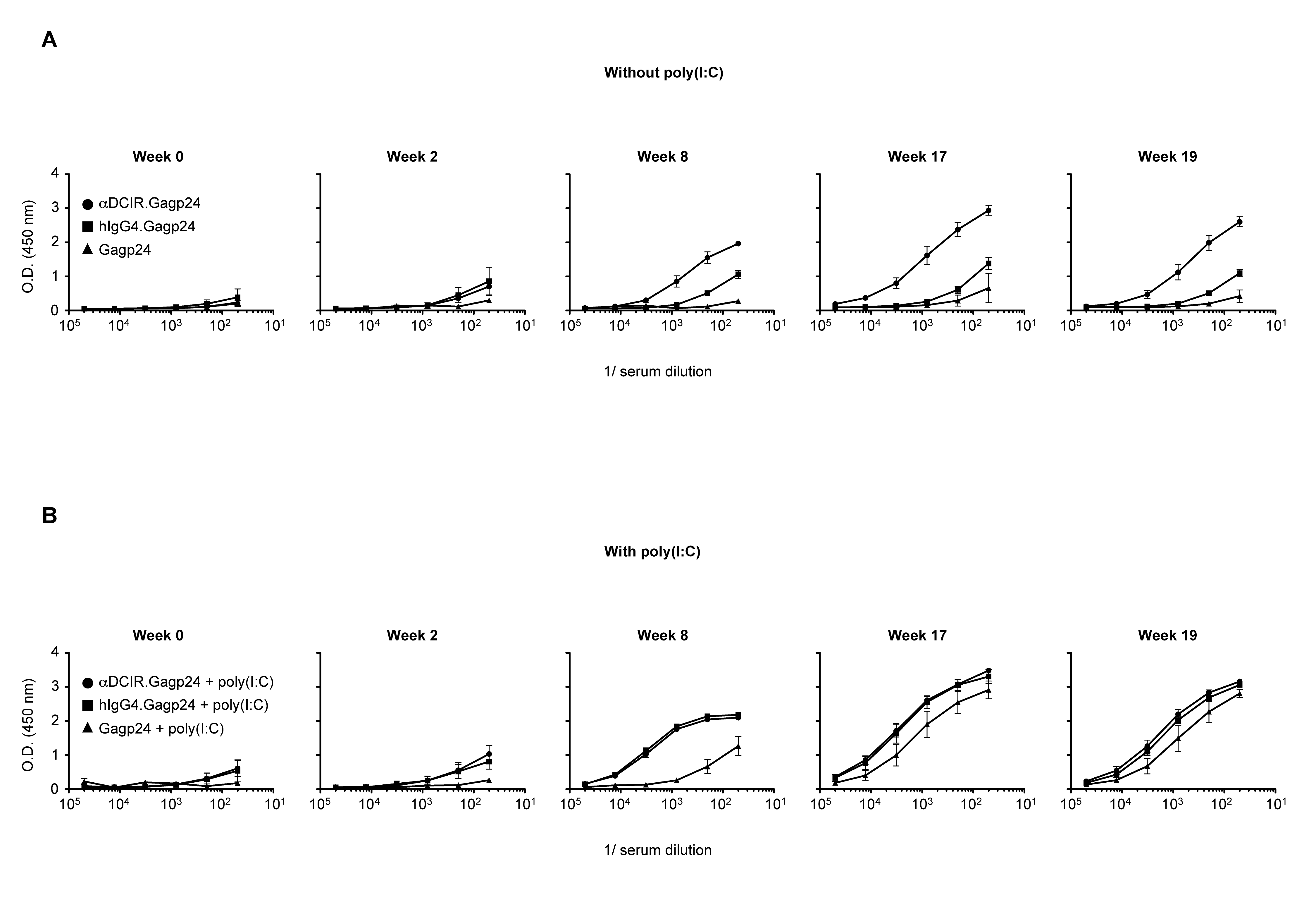

Supplement: S2 Fig — Animals were immunized i.d. three times at week 0, 6 and 15 with αDCIR.Gag24 or control hIgG4.Gagp24 or the molar equivalent of the Gagp24 protein with or without poly(I:C). HIV Gagp24-specific IgG antibody titers in serum were measured by ELISA at indicated time points post-immunization with αDCIR.Gagp24 or hIgG4.Gagp24 or Gagp24 without adjuvant (A) or with poly(I:C) (B). Data are presented as mean ± SEM. (TIF) [file pone.0135513.s002.tif]
